# Rapid discrimination of the native medicinal plant *Adenostemma lavenia* from its adulterants using PCR-RFLP

**DOI:** 10.7717/peerj.13924

**Published:** 2022-11-01

**Authors:** Kunchang Wu, Yunchen Liu, Bocheng Yang, Yenying Kung, Kaiwei Chang, Mengshiou Lee

**Affiliations:** 1 School of Pharmacy, College of Pharmacy, China Medical University, Taichung, Taiwan; 2 Department of Chinese Pharmaceutical Sciences and Chinese Medicine Resources, College of Chinese Medicine, China Medical University, Taichung, Taiwan; 3 Institute of Traditional Medicine, College of Medicine, National Yang Ming Chiao Tung University, Taipei, Taiwan; 4 Center of Traditional Medicine, Taipei Veterans General Hospital, Taipei, Taiwan; 5 Faculty of Medicine, School of Medicine, National Yang Ming Chiao Tung University, Taipei, Taiwan

**Keywords:** *Adenostemma lavenia*, Identification of origin, Molecular identification, Functional food

## Abstract

**Background:**

In Taiwan, the aerial part of *Adenostemma lavenia* (Al) is used in the form of herbal tea or in a folk remedy primarily to mitigate inflammatory conditions in the lungs and liver. Due to the excellent health benefits of Al against inflammation, it has become increasingly crucial and in great demand during the COVID-19 pandemic. However, Al has been found to be adulterated with *Wedelia biflora*, *Sigesbeckia orientalis*, and/or *Wedelia chinensis* because of similarities in appearance and vernacular names.

**Methods:**

This study aimed to develop a PCR-RFLP DNA molecular method for the authentication of Al. The restriction enzyme *Bsr*I was used according to the sequencing and alignment results of PCR products in the ITS2 regions of Al and its adulterants. Gel electrophoresis resulted in the clear separation of Al and its adulterants into two distinct categories.

**Results:**

In conclusion, the PCR-RFLP authentication method developed herein provides an easy, rapid, and accurate method to distinguish Al from its adulterants to assure user health and safety.

## Introduction

The medicinal plant *Adenostemma lavenia* (L.) Kuntze (Al) is known in Taiwan by its Chinese name “Xia-Tian-Ju (XTJ)” and grows widely in the tropical regions of Asia and the Pacific Islands (Cheng, Hufford & Doorenbos, 1979). The fruit of Al contains glands and mucus, which tends to stick to hands or clothes like glue, so Al is known colloquially in Chinese as “Ma-Zhi-Hu (MZH)”. Al is an herbaceous plant that is native to Taiwan and well cultivated in the cities of Yuanlin and Chiayi of Taiwan for applications in folk remedies and dietary supplements (Cheng, Hufford & Doorenbos, 1979; Lai et al., 2020).

The aerial part of Al is used in the form of herbal tea or in a medicinal cuisine to mitigate inflammatory conditions in the lungs and liver and to treat skin wounds and ulcers (Cheng, Hufford & Doorenbos, 1979; Prasad & Shyma, 2013; Prasad, Shyma & Raghavendra, 2013). According to previous literature, the main secondary compounds of Al are monoterpenes, sesquiterpenoids, *ent*-kaurane-type diterpenoids, triterpenes, and steroids (Cheng, Hufford & Doorenbos, 1979; Lin, 2013; Shimizu et al., 1990; Yang et al., 2007). Several reports have shown that the health benefits of Al are associated with its bioactive components, mainly *ent*-kaurane-type diterpenoids. The *ent*-kaurane-type diterpenoids, such as adenostemmoic acid B and *ent*-11 *α*-hydroxy-15-oxo-kaur-16-en-19-oic acid, have been reported to be the most important bioactive compounds in Al, and they have been shown to possess physiological activities that include those against cancer, inflammation, aging, and melanogenesis (Batubara et al., 2020; Hamamoto et al., 2020; Maeda et al., 2021; Shimizu et al., 1990). Moreover, the ethyl acetate fractions of Al extract have been reported to significantly alleviate the inflammatory symptoms of pneumonia in lipopolysaccharide-induced lung injury (Chen et al., 2019). Thus, Al features valuable beneficial functions as well as the feasibility of being used as a functional food for the prevention of and/or therapy for inflammatory conditions (Hamamoto et al., 2020).

As an herbal tea or in a dietary supplement, Al has been claimed to be beneficial to lung health, so it has become increasingly important and in great demand during the COVID-19 pandemic in Taiwan (Lai et al., 2020). Based on our previous field investigation, we found that Al is sometimes adulterated with three other species in Asteraceae that are similar in appearance and/or have vernacular names similar to those of Al: *Wedelia biflora* (L.) DC. (Wb), *Sigesbeckia orientalis* L. (So), and *Wedelia chinensis* (Osbeck) Merr. (Wc) (Lai et al., 2020). Such adulteration may impair the food or medication safety, health-promoting abilities, and consumer confidence in Al.

Although the health-benefitting properties and chemical components of Al have already been scientifically reported, there have not yet been any relevant authentication or quality control research reports on Al published. Phytochemical component analysis and various traditional identification methods, such as organoleptic, macroscopic, and microscopic identification techniques, not only rely deeply on a researcher’s expertise and experience but also cannot be applied alone to closely related species that possess similar chemical components and morphological structures (Han et al., 2016). Thus, DNA molecules are more desired and promising targets for developing an examination method for identifying herbal medicines. At present, a variety of DNA sequence-based species identification methods have been developed and applied, including random amplified polymorphic DNA (RAPD), amplified fragment length polymorphism (AFLP), and polymerase chain reaction-restriction fragment length polymorphism (PCR-RFLP) (Ha et al., 2002; Kaundun & Matsumoto, 2003). The aforementioned methods of DNA polymorphism analysis are species specific, cost-effective, rapid, and feasible for developing alternative assays to avoid adulteration (Abubakar et al., 2018; Yang et al., 2020; Zhu et al., 2018).

Adulteration of an herbal medicine can threaten the health of users (Han et al., 2016). To the best of our knowledge, there has not been a report that utilizes DNA molecular techniques to authenticate Al and separate it from its misused adulterants by means of DNA molecular markers in the ITS region of the nuclear rDNA. Thus, this study aimed to develop a simple, reliable, and accurate PCR-RFLP DNA molecular method for Al market supervision to assure the health of users in terms of safety and efficacy of the medicine. Eventually, it is hoped that this PCR-RFLP authentication method for Al will be validated by commercially available Al market samples from throughout Taiwan.

## Materials & Methods

### Plant materials

**Figure 1:**
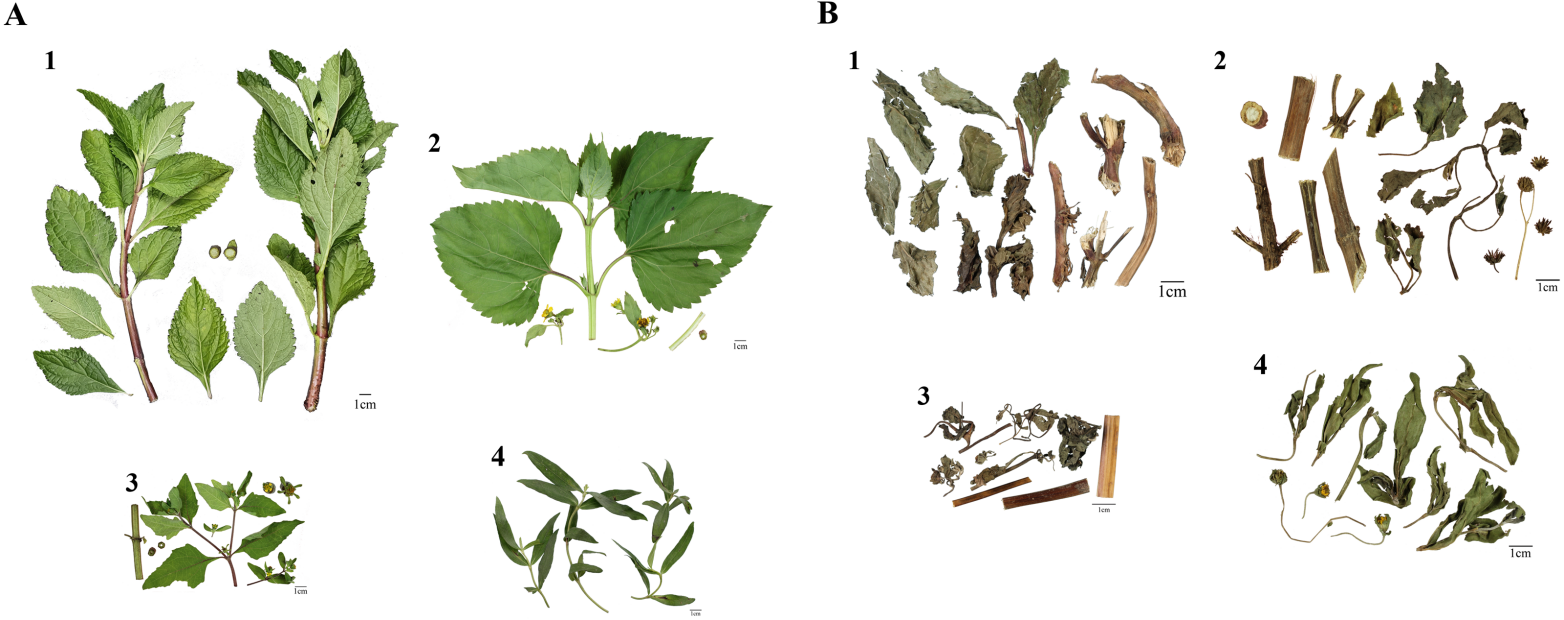
Morphological comparison of fresh plants (A) and dried herbal material (B) between *Adenostemma lavenia* (Al), *Wedelia biflora* (Wb), *Sigesbeckia orientalis* (So), and *Wedelia chinensis* (Wc). The numbers 1–4 represent Al, Wb, So, and Wc, respectively.

To obtain the reference DNA sequences for Al and its adulterants, viz., Wb, So, and Wc, all plants were collected and authenticated with ten batches of Al, three batches of Wb, two batches of So, and two batches of Wc, respectively, at different localities in Taiwan (Fig. 1, Table 1). For the specific investigation of Al market samples, the fourteen Al market samples were randomly purchased from herbal shops around Taiwan by referring to Al as XTJ or using the vernacular name MZH. All herbal samples were authenticated by Professor Yuan-Shiun Chang (China Medical University), and they were deposited in the collection at the School of Pharmacy at China Medical University.

### DNA extraction

Dried leaves from the authenticated samples of Al and its adulterants were collected and ground with liquid nitrogen into powder to allow for DNA extraction (Yang et al., 2020). Total DNA was isolated from each sample of homogenized plant tissue utilizing a Plant Genomic DNA Purification Kit (GeneMark, Atlanta, GA, USA) according to the manufacturer’s instructions. After DNA extraction, the concentrations of total genomic DNA were determined by a spectrophotometer (NanoDrop-1000; Thermo Fisher Scientific, Waltham, MA, USA), and each sample was stored at −20 °C.

### Phylogenetic tree analysis

The sequence regions of internal transcribed spacer 2 (ITS2), Rubisco large subunit (*rbcL*), and transfer RNA asparagine-like (*trnL*) of Al and its adulterants were used for sequence alignment by Vector NTI Advanced 11 software, and three different phylogenetic trees were constructed by MEGA-X software (Lorusso & Gemmellaro, 2021). All the sequences of Al and its three adulterants were available and obtained from the Genbank database of the National Center for Biotechnology Information (NCBI, https://www.ncbi.nlm.nih.gov/).

**Table 1:** Information on samples of Al and its adulterants collected from various localities in this study.

Scientific name	Collection locations	Abbreviation	Voucher number
*Adenostemma lavenia*	Taipei	Al-1	CMURX2020002
	Taichung	Al-2	CMURX2020005
	Taichung	Al-3	CMURX2020006
	Taichung	Al-4	CMURX2020007
	Chiayi	Al-5	CMURX2020008
	Chiayi	Al-6	CMURX2020009
	Taichung	Al-7	CMURX2020010
	Hsinchu	Al-8	CMURX2020012
	Yunlin	Al-9	CMURX2020013
	Taichung	Al-10	CMURX2020014
*Wedelia biflora*	Hualian	Wb-1	CMURX2020001
	Taichung	Wb-2	CMURX2020015
	Changhua	Wb-3	CMURX2020016
*Sigesbeckia orientalis*	Changhua	So-1	CMURX2020017
	Taichung	So-2	CMURX2020018
*Wedelia chinensis*	Taichung	Wc-1	CMURX2020019
	Chiayi	Wc-2	CMURX2020020

### Polymerase chain reaction (PCR) and electrophoresis

First, the ITS2 regions of Al and its three adulterants were amplified by ITS-2F and ITS-3R primers using genomic DNA from various Asteraceous species as the template DNA (Yang et al., 2020). The two oligonucleotide primers ITS-2F (5′-ATGCGATACTTGGTGTGAAT-3′) and ITS-3R (5′-GACGCTTCTCCAGACTACAAT-3′) were designed and stemmed from the 18S and 26S rDNA sequences of Al and its three adulterants (Yang et al., 2020). DNA amplification was performed in a 25 µL reaction mixture including 12.5 µL *Taq* DNA polymerase 2 × Master Mix RED (Ampliqon A/S, Odense, Denmark), 9.5 µL double distilled water, 1 µL each of 10 µM ITS-2F and ITS-3R, and 1 µL template DNA, which was diluted with double-distilled water until its concentration became one-tenth of its original value. The PCR conditions were 95 °C for 3 min; followed by 30 cycles at 95 °C for 30 s, 55 °C for 30 s, and 72 °C for 30 s; and a final extension for 5 min. The amplified products were resolved using 2.2% agarose gels that contained 0.1 µL/mL GelRed Nucleic Acid Gel Stain (Biotium, Fremont, CA, USA) in 1 × Tris-Acetate-EDTA buffer (TAE) with a 100-bp ladder as a marker.

### PCR product sequencing and restriction fragment length polymorphism (RFLP) analysis

Each PCR product amplified by ITS-2F and ITS-3R was sequenced and aligned by Vector NTI Advanced 11 software, which was used to statistically analyze the percentage similarities of Al with its three adulterants. In addition, the sequences of Al and its adulterants were aligned by the Clustal Omega (https://www.ebi.ac.uk/Tools/msa/clustalo/) webserver (Sievers & Higgins, 2021). For these PCR products, sequence restriction maps were established and competed against one another on the NEBcutter V2.0 (http://nc2.neb.com/NEBcutter2/) webserver (Gargouri, Moalla & Kacem, 2021). One restriction enzyme, *Bsr*I, was selected and predicted to differentiate Al from the other three Asteraceous adulterants. Furthermore, we added a commercial vector, pcDNA3.1(+), as a positive control. The two oligonucleotide primers pcDNA-F (5′-CTGAATGAACTGCAGGACGA-3′) and pcDNA-R (5′-GGCGGTGGAATCGAAATCTC-3′) in the vector were designed to amplify a selected sequence containing a cutting site for *Bsr*I. Following the instructions from the manufacturer (New England Biolabs, Ipswich, USA), a 50 µL PCR-RFLP reaction mixture was prepared that contained 3 µL PCR product, 1 µL restriction enzyme, *Bsr*I, 41 µL double distilled water, and 5 µL NEBuffer. Next, all the mixtures, including those for Al and its three adulterants as well as the positive control, were incubated at 65 °C for 15 min. Finally, the RFLP products were determined using 2.2% agarose gels that contained 0.1 µL/ml GelRed nucleic acid gel stain (Biotium) in 1 × TAE with a 100-bp ladder as a marker.

### Application of the PCR-RFLP assay for the survey of authenticity of *A. lavenia* from markets

Fourteen batches of commercial herbal samples of Al were collected as described above. DNA was extracted from each sample and determined by spectrophotometry. Then, PCR-RFLP was performed to discriminate between Al and its adulterants. The final results were obtained by DNA electrophoresis on agarose gels.

## Results

### Morphological examination of plant specimens

Al and its three adulterants, Wb, So, and Wc, collected from various localities, were used, and their morphologies were compared, as illustrated in Fig. 1. Based on morphological appearance, the leaves and stems of Wc showed significant differences from those of Al, Wb, and So (panel 4 of Fig. 1A). There were few morphological differences in leaves or stems among Al, Wb, and So (panels 1, 2, and 3 of Fig. 1A, respectively). Moreover, the dried forms of Al, Wb, So, and Wc that were collected from various herbal shops showed no significant variation in appearance among them (panels 1, 2, 3, and 4 of Fig. 1B). Based on the morphological examination and comparison described above, Al is prone to be adulterated with other species similar in appearance, which alerts us to be concerned about the issue of Al adulteration with other species.

### Phylogenetic tree analysis for DNA barcode selection

**Figure 2:**
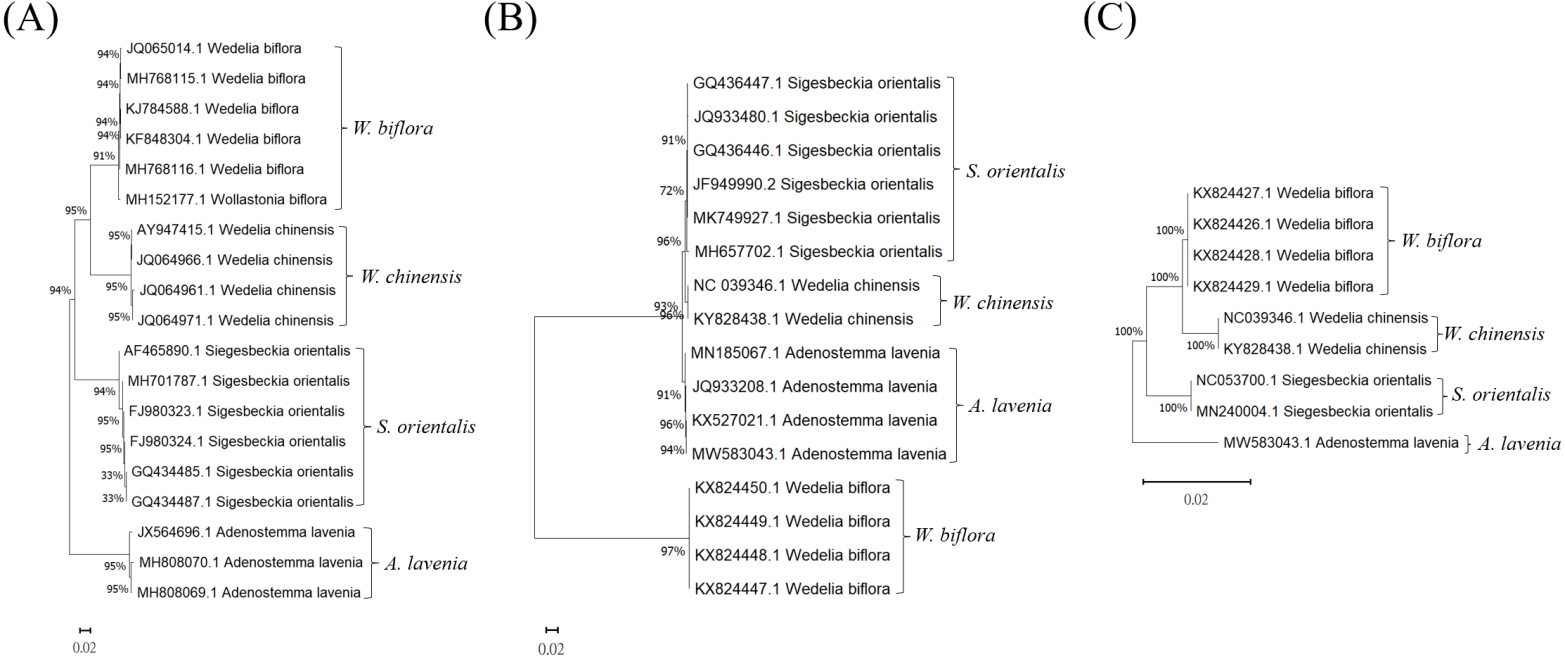
Phylogenetic trees of Al, Wb, So, and Wc produced for reference sequences in the (A) ITS2, (B) *rbcL*, and (C) *trnL* regions. The DNA sequences of different species were compared with the GenBank database of the National Center for Biotechnology Information (NCBI). The percentage on the node for each branch is a bootstrap score from 1,000 replicates.

**Figure 3:**
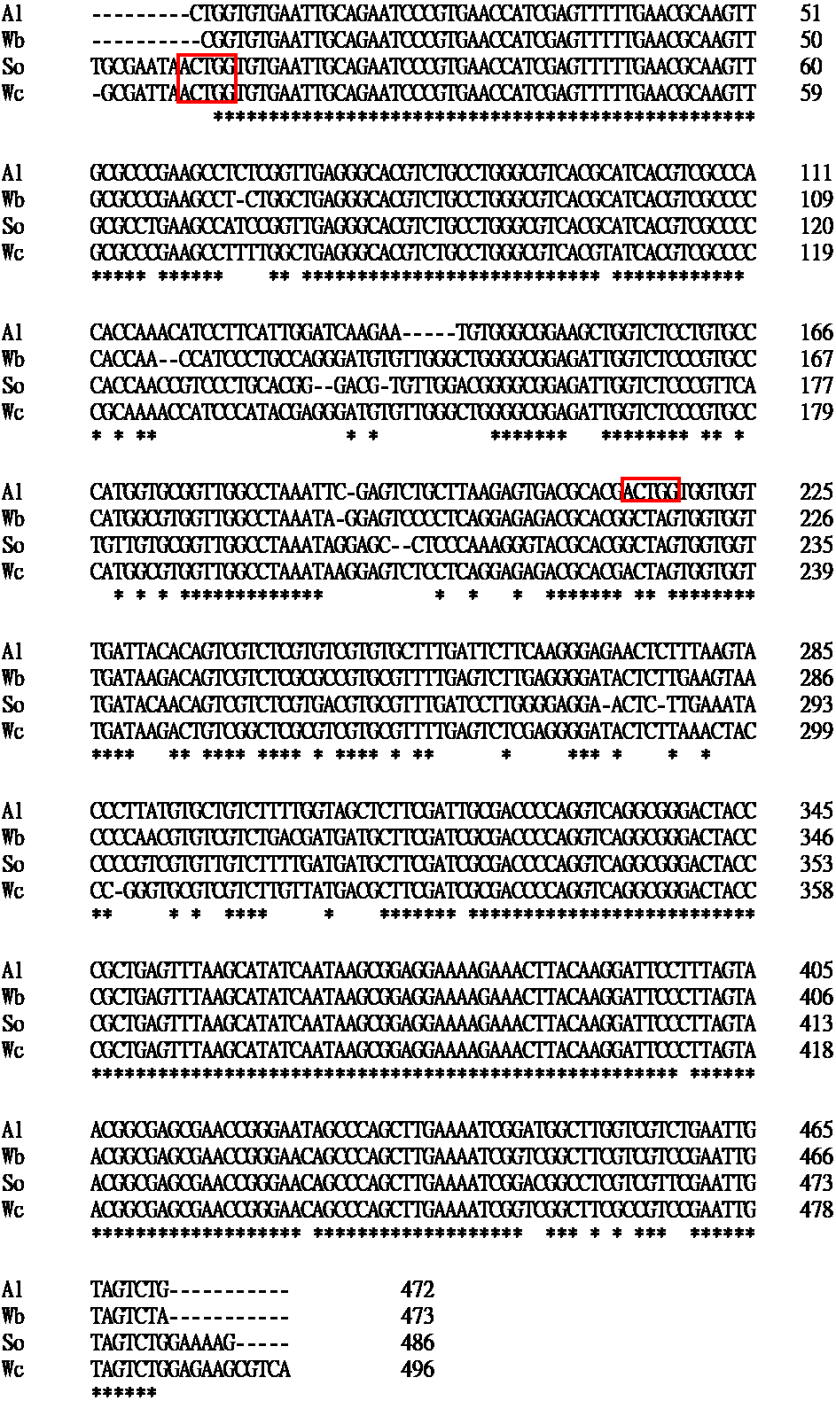
ITS2 sequence comparison between Al and its adulterants. The *Bsr* I recognition site is marked with a red box. An asterisk (*) represents an aligned nucleotide that is identical in all sequences. A hyphen (-) indicates a gap in the aligned sequence.

To select a suitable DNA barcode for the molecular authentication of Al and for distinguishing it from its adulterants, three commonly used potential DNA barcodes, ITS2, *rbcL* and *trnL*, were chosen to build phylogenetic trees to evaluate the sequences for their discriminatory power (Fig. 2). As illustrated in Fig. 2A, the phylogenetic analysis of the ITS2 regions was accomplished by the neighbor-joining (NJ) method. This ITS2-based phylogenetic analysis of Al and its adulterants formed four distinct clades (Fig. 2A). Furthermore, for *rbcL* and *trnL*, the phylogenetic trees produced also exhibited a similar discriminatory pattern in forming distinct clades for Al and its adulterants (Figs. 2B & 2C). These results indicate that ITS2, *rbcL*, and *trnL* could all be used for Al authentication. However, regarding the comparison among the genetic distances in ITS2-, *rbcL*- and *trnL*-based phylogenetic trees, the ITS2-based phylogenetic tree demonstrated greater genetic distances between the species than did the *rbcL*- and *trnL*-based phylogenetic trees. In further examining the sequence divergence in the ITS2 tree, 85–94% sequence similarities were found between Al and its three adulterants (Fig. 3 and Table 2). This is reflected in the sequence divergence in the ITS2 tree, which ranged from 6–15% between Al and its three adulterants. This strongly supports the credibility of this NJ tree, and the range implies that Al shows sufficient sequence divergence on the ITS2 tree, which increases the possibility of successful Al authentication once a rapid identification assay is established. In summation, ITS2 can be concluded to hold higher potential as a molecular marker than do *rbcL* or *trnL* to authenticate Al and distinguish it from its adulterants.

**Table 2:** Analysis of sequence similarities for ITS2 regions between Al and its adulterants.

Species	Al	Wb	So	Wc
Al		85%	86%	86%
Wb			90%	88%
So				94%
Wc				

### Development of a PCR-RFLP assay for *A. lavenia* authentication

**Figure 4:**
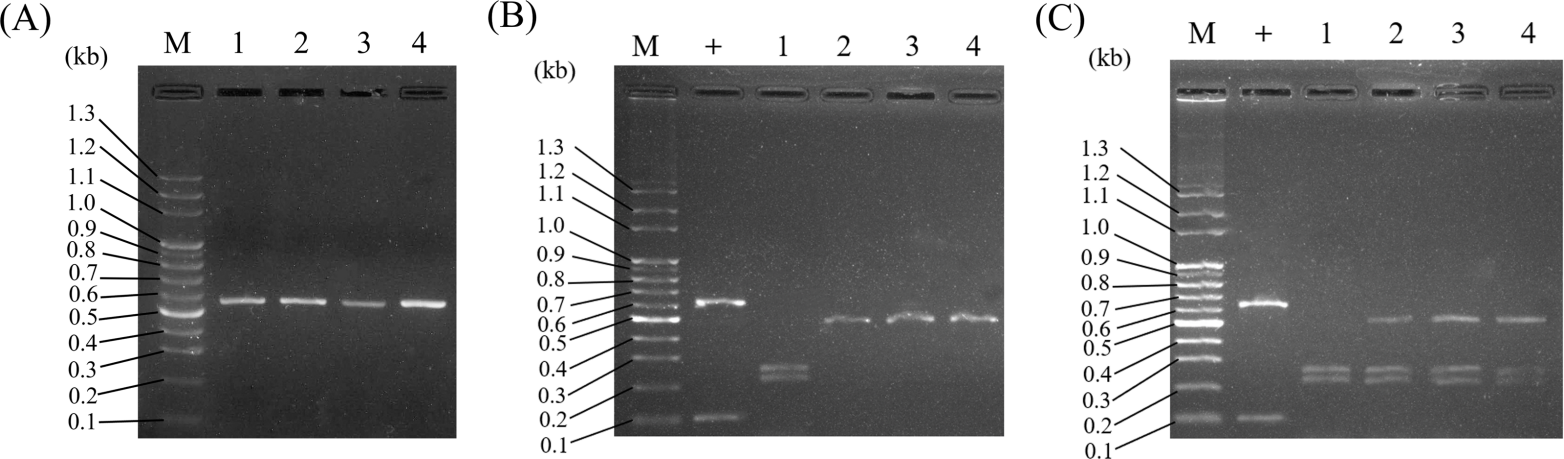
Establishment of PCR-RFLP pattern for Al authentication and discrimination of Al and its adulterants. (A) PCR-amplified ITS2 was revealed for the species Al, Wb, So, and Wc. Lane M, DNA marker; lanes 1–4, Al, Wb, So, and Wc, respectively. (B) PCR-RFLP patterns for Al authentication were produced in agarose gel. PCR-amplified ITS2 was digested by *Bsr* I. Lane M, DNA marker; +, positive control; lanes 1–4, Al, Wb, So, and Wc, respectively. (C) PCR-RFLP analysis for Al authentication and adulteration. Samples of Al mixed with its adulterants were examined by RFLP. Lane M, DNA marker; +, positive control; lane 1, Al; lanes 2–4, Al mixed with Wb, So, and Wc, respectively.

Because it was concluded that ITS2 is a useful molecular marker for authenticating Al, ITS2 was herein used as a specific DNA marker for the development of a PCR-RFLP assay for the convenient authentication of Al plants. As illustrated in Fig. 4A, PCR amplified ITS2 fragments, which were successfully produced and sequenced using ITS2 universal primers when the genomic DNA of Al, Wb, So, and Wc was used as templates. After sequencing, four different DNA sizes, 472 bp, 473 bp, 486 bp and 496 bp, which corresponded to Al, Wb, So and Wc, respectively, were resolved in agarose gel (Fig. 4A & Table 3). Among these, various ITS2 sequences showed a similar pattern of DNA size. It was difficult to distinguish Al from its adulterants instantly, but this could be accomplished by polymerase chain reaction (Fig. 4A). After predicting the restriction profiles for ITS2 by NEBcutter (http://nc2.neb.com/NEBcutter2/), only one restriction enzyme cutting site, *Bsr*I, was recognized in Al, So, and Wc (Fig. 3). There were no *Bsr*I cutting sites in ITS2 of Wb (Fig. 3). Thus, when *Bsr*I was used to digest ITS2 from Al, Wb, So, and Wc, two DNA fragments (253 bp and 219 bp) produced from the Al sample, two fragments (472 bp and14 bp) produced in the So, and two fragments (483 bp and 13 bp) produced in the Wc were revealed, which made it easy to discriminate Al and its adulterants into two distinct patterns (Fig. 4B & Table 3). It is worth noting that the restriction profiles with *Bsr*I of the three adulterants, Wb, So, and Wc, demonstrated no significant difference in DNA banding patterns after electrophoresis (Fig. 4B, lanes 2–4). The ITS2 DNA from Wb was not affected by *Bsr*I digestion. Simultaneously, for the validity of digestion, we chose a 700-bp sequence containing a cutting site for *Bsr*I from a commercial vector, pcDNA3.1(+), to provide a positive control for the experiment. The result showed two DNA fragments (600 bp and 100 bp) for DNA banding patterns after electrophoresis. This result effectively demonstrated that the restriction enzyme was fully functioning. Furthermore, the typical RFLP pattern of Al was altered when the Al sample was mixed with one of three adulterants (Fig. 4C). Taken together, through the experimental investigation of restriction profiles, a PCR-RFLP analysis for Al authentication was established. This analysis could rapidly authenticate Al and discriminate its adulterants easily when adulteration of Al had occurred.

**Table 3:** The DNA sequence lengths of predicted restriction fragments cleaved from the PCR-amplified ITS2 regions of Al and its adulterants.

Scientific name	Length (bp)	
	ITS2^a^	Cleaved ITS2^b^
*Adenostemma lavenia*	472	253/219
*Wedelia biflora*	473	473
*Sigesbeckia orientalis*	486	472/14
*Wedelia chinensis*	496	483/13

**Notes.**

a The lengths of ITS2 region of *Adenostemma lavenia* and its three adulterants were amplified by ITS-2F and ITS-3R primers.

b Predicted restriction fragments of different sizes after *Bsr* I digested.

### Application of PCR-RFLP assays for authenticated Al and its adulterants in different localities

**Figure 5:**
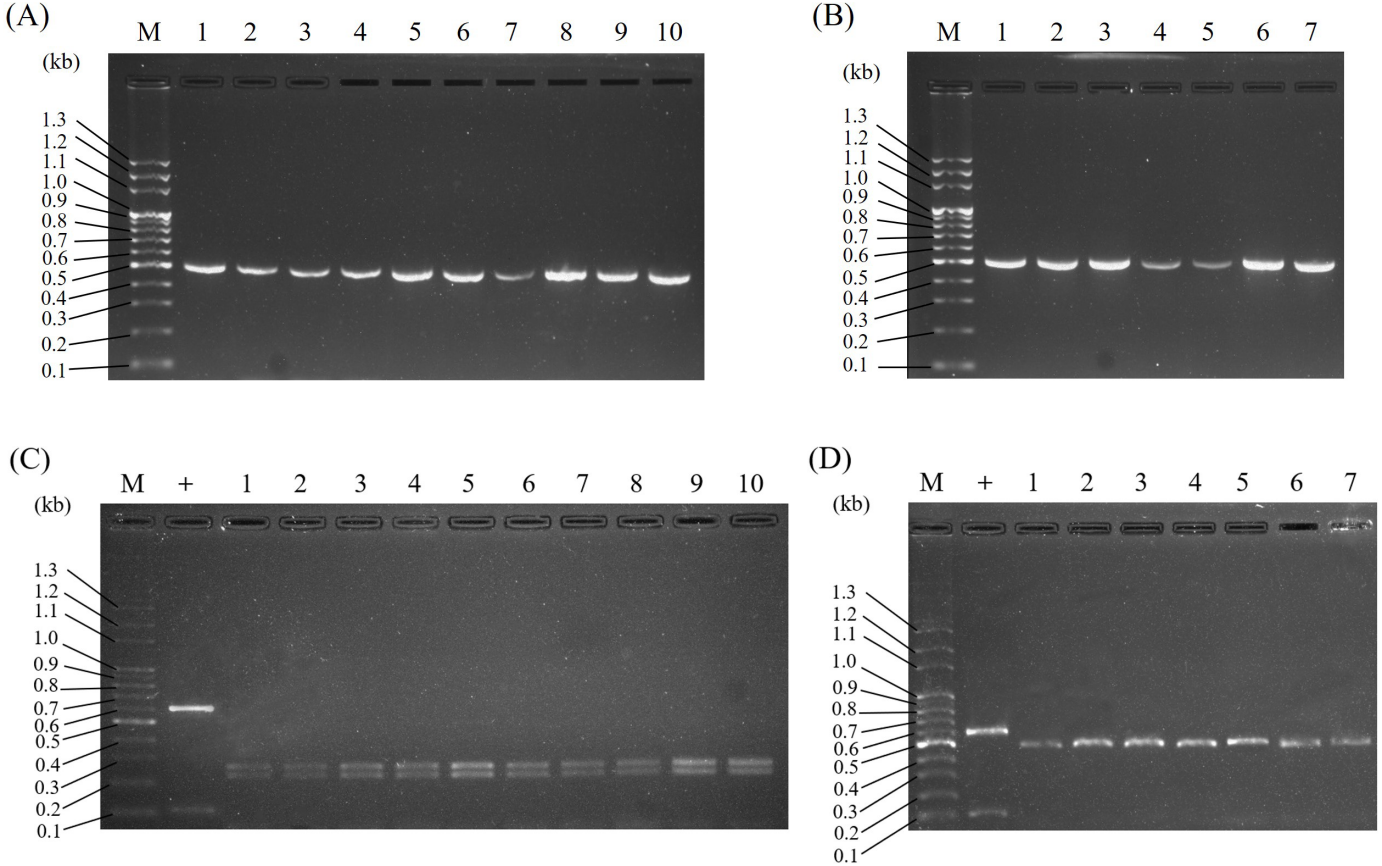
Application of PCR-RFLP analysis for the authentication of Al and its adulterants collected from various localities. The ITS2 regions of the four species Al, Wb, So, and Wc were amplified by PCR (A, B). RFLP analysis was conducted by *Bsr* I following PCR to differentiate Al and its adulterants (C, D). In panels (A) and (C), lane M indicates a DNA marker, + indicates a positive control, and lanes 1–10 indicate Al samples collected from various localities. For panels (B) and (D), lane M indicates a DNA marker, + indicates a positive control, lanes 1–3 indicate Wb samples collected from three different localities, lanes 4–5 indicate So samples collected from two different localities, and lanes 6–7 indicate Wc samples collected from two different localities.

To assure the applicability of the PCR-RFLP technique for DNA molecular identification of Al, ten authenticated Al and seven adulterated samples, including Wb, So, and Wc samples collected from various localities, were tested. As shown in Fig. 5, the ITS2 sequences of all the samples were amplified by PCR (Figs. 5A & 5B), and the DNA sequence lengths of the PCR-amplified ITS2 were expected to be the same as those in Fig. 4A. After *Bsr*I digestion, all of the samples were cleaved, and a typical RFLP pattern for Al and Al adulterants was produced. These data suggest that the PCR-RFLP method could be applied to Al sample identification in various localities throughout Taiwan.

### Application of the PCR-RFLP assay for the investigation of adulterants in Al samples from herbal markets

**Figure 6:**
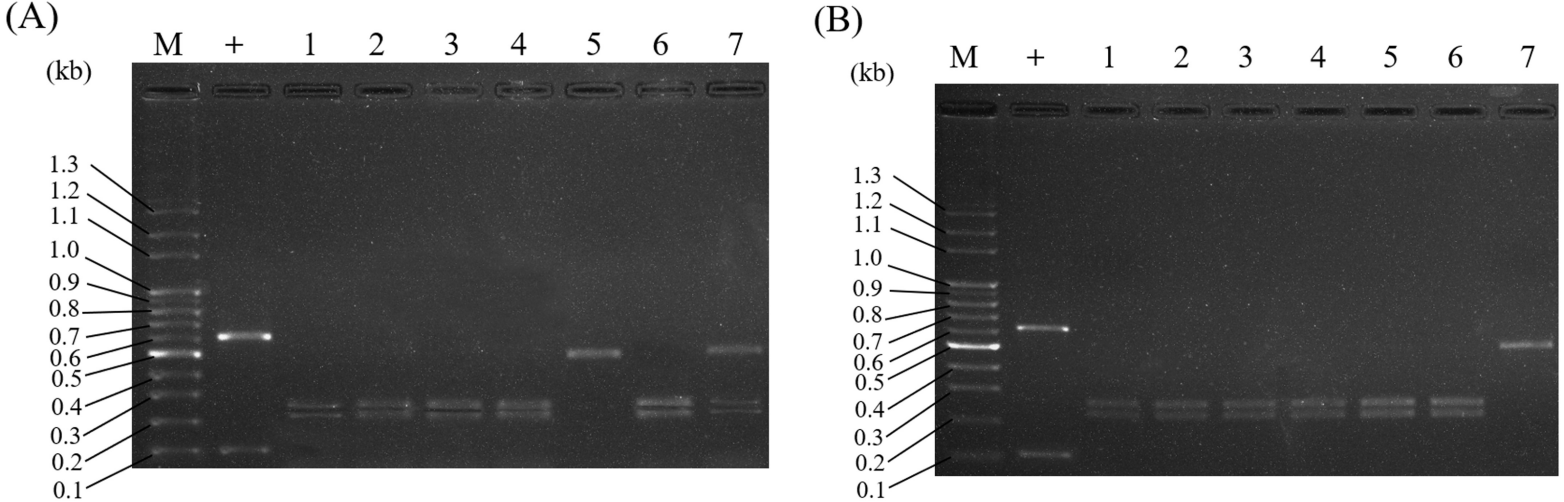
Application of PCR-RFLP analysis for the investigation of Al adulteration in Al samples from herbal markets. A total of fourteen commercial Al samples were collected from various herbal markets in Taiwan. ITS2 regions of all samples were subjected to RFLP analysis. The collected Al samples in (A) and (B) represent the samples purchased from different herbal vendors. Lane M in (A) and (B) indicates the DNA marker; + in (A) and (B) indicates the positive control.

To investigate Al adulteration, commercial Al herbal plants purchased from various herbal markets were used for authentication and identification by the PCR-RFLP assay. There were a total of fourteen Al samples. As expected, the identification of the market samples clearly separated Al and its adulterants into two different categories. Among the fourteen commercially purchased Al samples, most were certified as authentic samples of Al by PCR-RFLP. There were three of the samples claimed to be Al that were actually adulterated (lanes 5 and 7 in Fig. 6A, lane 7 in Fig. 6B). The authentic samples of Al demonstrated DNA fragments of lengths 253 bp and 219 bp in a typical Al RFLP pattern. On the other hand, the adulterants’ patterns are shown in lanes 5 and 7 in Fig. 6A and lane 7 in Fig. 6B. These results indicate that 21.4% (3/14) of the Al samples purchased randomly in herbal markets were adulterated. In summary, this established PCR-RFLP assay can indeed be applied for Al identification, and our Al authentication results showed that approximately 21.4% of the samples from herbal markets were adulterated.

## Discussion

Differentiating Al from its adulterants is a critical issue because in Taiwan, Al is not only a native cultivated plant but also plays a crucial role in herbal tea, possessing value for lung protection and treatment (Cheng, Hufford & Doorenbos, 1979; Lin, 2013). Furthermore, the bioactive diterpene ingredients of Al have significant anti-inflammatory effects in pneumonia that may result from COVID-19 (Chen et al., 2019; Maeda et al., 2021). With the aforementioned health-promoting abilities of Al in mind, establishing an accurate appraisal of the authenticity of Al is essential. In addition, it should be noted that Al in Japan is included on the Red List as an endangered species, while Al in Taiwan is well cultivated (Maeda et al., 2021). This places even greater emphasis on the importance of discriminating between Al and its adulterants.

Among the variety of authentication methods, PCR-RFLP is a useful and popular tool for nonprofessional users, viz., traditional drug producers and managers, customs officers, and forensic specialists (Pennisi, 2007; Xin et al., 2015). Factors influencing a successful DNA molecular method include universal priming sites, a high level of amplification, an excellent DNA sequencing result, and enough disparity to identify the sample(s) (Parvathy et al., 2015). One of the key points of this study was to find suitable DNA loci to discriminate Al from three other species of plants often confused with it. In the present study, a phylogenetic evolutionary tree was built for Al and its adulterants using ITS2, *rbcL*, and *trnL* for comparison. The best one was found to be ITS2, which was supported by the values of nodes on almost all exceeding 90%, indicating a reliable evolutionary relationship (Alaklabi et al., 2021). Moreover, in previous research (Chen et al., 2010; Hollingsworth, Graham & Little, 2011; Xiong et al., 2016), ITS2 has been shown to be the best locus among seven different candidate barcodes (*psbA-trnH*, *matK*, *rbcL*, *rpoC1*, *ycf5*, ITS2, and ITS) from medicinal plant species. ITS2 succeeded in the identification of species with 92.7% accuracy. Therefore, we chose the ITS2 region to serve as the main barcode for distinguishing Al from its adulterants.

This study was the first report to develop a PCR-RFLP assay for identifying Al and its adulterants as well as distinguishing between them by means of DNA barcoding. In past reports, research concerning the molecular biology of Al has been rare (Xia & Li, 2021). All four species used in this study have previously been detected to acquire their composition and the certain active ingredients they contain for the promotion of good health (Gao et al., 2018; Hamamoto et al., 2020; Shimizu et al., 1990; Yang et al., 2007). Some past studies have also utilized genomic ribosomal DNA sequences to differentiate interspecific relationships and build NJ trees with other plants (Gao et al., 2018). However, no one thus far has completed an in-depth exploration of a rapid and easy method to judge the authenticity of Al samples. Direct DNA sequencing is accurate in analyzing Al and the species with which it is confused. However, this technique is inefficient and time-consuming (Lin & Hwang, 2007). In contrast, the PCR-RFLP method used in this study is much more cost-effective, simple, and convenient, provided there is sufficient quantity and quality of DNA products for species identification. Furthermore, according to the results of this study, PCR-RFLP is appropriate for authenticating commercial samples of Al and its adulterants because the DNA barcodes found apparently demonstrated its discriminatory power (Ghatak, Muthukumaran & Nachimuthu, 2013). Nevertheless, this method does have a restriction in that small fragments would hardly be seen owing to the insensitivity of gel electrophoresis. In general, fragments smaller than 80 bp are difficult to identify on agarose gels (Wolf, Rentsch & Hübner, 1999). Although this disadvantage exists, in this study, we could still clearly detect the differences between Al and its adulterants.

With a certain testing method of quality control for Al needed, we found that PCR-RFLP can be widely used to investigate the authenticity of the Al found in herb markets all over Taiwan. On the other hand, in addition to the development of the PCR-RFLP method, we also performed an interesting modified experiment with those Al samples containing adulterants, which may include two or more species. In our study, with the same identification material, the pure Al samples could still be easily discriminated from the impure. The adulterated mixtures were also clearly detected because of the pattern of three fragments of different sizes. This phenomenon reaffirms the validity of the PCR-RFLP method for determining the purity of Al.

Toward the end of the period when we were using the material for the discrimination of Al from its potential adulterants, we randomly collected fourteen commercial samples of Al from herbal shops in various localities in Taiwan. Through the evidence obtained from the PCR-RFLP method, our results demonstrated that most of these samples were real Al; nevertheless, there were three samples that contained adulterants. Among these three, two consisted of other plants that had been substituted for Al, and one of them contained Al mixed with another plant. This phenomenon represents the necessity of the development of a rapid method for identification of authentic Al.

In the research on commercial herbal samples of Al, we successfully identified Al and its adulterants in fourteen collected market Al samples. The results of our examination indicate that PCR-RFLP is useful for DNA sequence analysis and can easily and precisely discriminate between Al and its adulterants. However, the disappearance of small DNA fragments was an imperfect aspect of this experiment. This problem may be addressed by the technique of capillary electrophoresis (CE). With typical CE, the detection of DNA fragments will be more accurate and provide better patterns for identification. In brief, in terms of the reports that Al is a threatened species in some places (Maeda et al., 2021), economical constraints and the difficulty of obtaining it may incentivize traders to replace it with cheaper substitutions and sell the adulterated product as if it were Al (Kool et al., 2012; Xin et al., 2015). Moreover, with the domestic food and drug safety situation gradually becoming more stringent, manufacturers and individuals have turned their attention from substitution to “illegal addition” and “process control” (Siddiqui et al., 2021; Xin et al., 2015). For this reason, raw material quality control has become a kind of security assurance for society. On the other hand, Al plays a vital role in the herbal market of Taiwan. However, the lack of ability to verify the authenticity of Al has been a vital issue.

## Conclusions

This work utilized the method of PCR-RFLP to discriminate samples of Al to determine which were pure and which were adulterated. With the PCR-RFLP method, an excellent investigatory tool is available. Finally, this study contributes to related research regarding Al and quality control as well as the security of consumer health.

##  Supplemental Information

10.7717/peerj.13924/supp-1 Supplemental Information 1 Primers used for amplification ofthe ITS2 barcodes of *Adenostemma lavenia* and its adulterants, viz., *Wedelia biflora*, *Sigesbeckia orientalis*, and *Wedelia chinensis*

10.7717/peerj.13924/supp-2 Supplemental Information 2 ITS2 barcode DNA sequences of the standard samples of *Adenostemma lavenia* and its adulterants, viz., *Wedelia biflora*, *Sigesbeckia orientalis*, and *Wedelia chinensis* To establish the standard ITS2 barcode DNA sequences for *Adenostemma lavenia* and its three adulterants, we collected four authenticated samples for further DNA sequencing.

## Additional Information and Declarations

### DNA Deposition

The following information was supplied regarding the deposition of DNA sequences: The ITS2 DNA sequences of *Adenostemma lavenia*, *Wedelia biflora*, *Sigesbeckia orientalis*, and *Wedelia chinensis* described here are available at GenBank: OM095431.1, OM095434.1, OM095435.1, and OM095436.1.

### Data Availability

The following information was supplied regarding data availability: All the raw data of ITS2 sequences of *Adenostemma lavenia* and its three adulterants viz *Wedelia biflora* (L.) DC., *Sigesbeckia orientalis* L., and *Wedelia chinensis* (Osbeck) Merr. are available in the Supplementary File.
