# Supplementary material for: Rapid discrimination of the native medicinal plant Adenostemma lavenia from its adulterants using PCR-RFLP
Source: PeerJ. 2022 Nov 1;10:e13924. doi: 10.7717/peerj.13924 (PMC9635354; doi:10.7717/peerj.13924)
Supplement: Supplemental Information 1 [file peerj-10-13924-s001.docx]

Table S1. Primers used for amplification of the barcodes ITS2

| **Primer pairs** | **Sequence** | ***Locus*** | **Annealing temperature** | **Reference** |
| --- | --- | --- | --- | --- |
| ITS-2F | ATGCGATACTTGGTGTGAAT | ITS2 | 60°C | Cheng et al. (2015) |
| ITS-3R | GACGCTTCTCCAGACTACAAT |  |  |  |

**References**

**Cheng T, Xu C, Lei L, Li C, Zhang Y, Zhou S. 2015**. Barcoding the kingdom Plantae: new PCR primers for ITS regions of plants with improved universality and specificity. Molecular Ecology Resources **16(1)**:138–149.
