# Supplementary material for: Rapid discrimination of the native medicinal plant Adenostemma lavenia from its adulterants using PCR-RFLP
Source: PeerJ. 2022 Nov 1;10:e13924. doi: 10.7717/peerj.13924 (PMC9635354; doi:10.7717/peerj.13924)
Supplement: Supplemental Information 2 — To establish the standard ITS2 barcode DNA sequences for Adenostemma lavenia and its three adulterants, we collected four authenticated samples for further DNA sequencing. [file peerj-10-13924-s002.docx]

> *Adenostemma lavenia* (L.) Kuntze, ITS2 intergenic spacer; voucher CMURX2020002.

TGGTGTGAATTGCAGAATCCCGTGAACCATCGAGTTTTTGAACGCAAGTTGCGCCCGAAGCCTCTCGGTTGAGGGCACGTCTGCCTGGGCGTCACGCATCACGTCGCCCACACCAA-ACATCC--TTCATTGGA--TCAAGAATGTGGGCGGAAGCTGGTCTCCTGTGCCCATGGTGCGGTTGGCCTAAATT-CGAGTCTGCTTAAGAGTGACGCACGACTGGTGGTGGTTGATTACACAGTCGTCTCGTGTCGTGTGCTTTGATTCTTCAAGGGAGAACTCTTTAAGTA-CCCTTATGTGCTGTCTTTTGGTAGCTCTTCGATTG

> *Wedelia biflora* (L.) DC., ITS2 intergenic spacer; voucher CMURX2020001.

CGGTGTGAATTGCAGAATCCCGTGAACCATCGAGTTTTTGAACGCAAGTTGCGCCCGAAGCCTCT-GGCTGAGGGCACGTCTGCCTGGGCGTCACGCATCACGTCGCCCCCACCAA-CCATCCC-TGCCAGGGATGTGTTGGGCTGGGGCGGAGATTGGTCTCCCGTGCCCATGGCGTGGTTGGCCTAAATA-GGAGTCCCCTCAGGAGAGACGCACGGCTAGTGGTGGTTGATAAGACAGTCGTCTCGCGCCGTGCGTTTTGAGTCTTGAGGGGA-TACTCTTGAAGTAACCCCAACGTGTCGTCTGACGATGATGCTTCGATCG

> *Sigesbeckia orientalis* L., ITS2 intergenic spacer; voucher CMURX2020017.

TGGTGTGAATTGCAGAATCCCGTGAACCATCGAGTTTTTGAACGCAAGTTGCGCCTGAAGCCATCCGGTTGAGGGCACGTCTGCCTGGGCGTCACGCATCACGTCGCCCCCACCAA-CCGTCCC-TGCACGGGACGTGTTGGAC-GGGGCGGAGATTGGTCTCCCGTTCATGTTGTGCGGTTGGCCTAAATA-GGAGCCTCC-CAAAGGGTACGCACGGCTAGTGGTGGTTGATACAACAGTCGTCTCGTGACGTGCGTTT-GATCCTTGGGGAGG-AACTCTTGAAATA-CCCCGTCGTGTTGTCTTTTGATGATGCTTCGATCG

> *Wedelia chinensis* (Osbeck) Merr., ITS2 intergenic region; voucher CMURX2020019.

TGGTGTGAATTGCAGAATCCCGTGAACCATCGAGTTTTTGAACGCAAGTTGCGCCCGAAGCCTTTTGGCTGAGGGCACGTCTGCCTGGGCGTCACGTATCACGTCGCCCCCGCAAAACCATCCCATACGAGGGATGTGTTGGGCTGGGGCGGAGATTGGTCTCCCGTGCCCATGGCGTGGTTGGCCTAAATAAGGAGTCTCCTCAGGAGAGACGCACGACTAGTGGTGGTTGATAAGACTGTCGGCTCGCGTCGTGCGTTTTGAGTCTCGAGGGGA-TACTCTTAAACTA-CCCGGGTGCGTCGTCTTGTTATGACGCTTCGATCG
